# Summary of the proceedings of the International Forum 2017: “Position of interventional radiology within radiology”

**DOI:** 10.1007/s13244-018-0594-5

**Published:** 2018-02-23

**Authors:** European Society of Radiology (ESR)

**Affiliations:** European Society of Radiology (ESR), Neutorgasse 9/2, 1010 Vienna, Austria

**Keywords:** Interventional radiology, Radiology, Education, Turf battles

## Abstract

**Abstract:**

The International Forum is held once a year by the ESR and its international radiological partner societies with the aim to address and discuss selected topics of global relevance in radiology. In 2017, the issue of the position of interventional radiology (IR) within radiology was analysed. IR is expanding because of the increased patient demand for minimally invasive therapies performed under imaging guidance, and its success in improving patient outcomes, reducing in-hospital stays, reducing morbidity and mortality of treatment in many organs and organ-systems. Despite the many successes of IR, public awareness about it is quite low. IR requires specific training and, in most countries, the majority of interventional radiologists do not dedicate their time completely to IR but perform diagnostic radiology investigations as well. Turf battles in IR are common in many countries. To preserve and keep IR within radiology, it is necessary to focus more on direct and longitudinal patient care. Having beds dedicated to IR within radiology departments is very important to increase clinical involvement of interventional radiologists. IR procedures fit perfectly within “value-based healthcare”, but the metrics have to be developed.

**Main messages:**

• *IR should stay a prominent subspecialty within radiology.*

• *Dedicated IR training pathways are mandatory.*

• *Measures to increase recruitment of young doctors to IR and to increase public awareness of IR are needed.*

• *Beds dedicated to IR within radiology departments are important in order to increase clinical involvement of interventional radiologists.*

## Introduction

The International Forum (formerly “International Summit”) was established by the European Society of Radiology (ESR) in order to intensify the collaboration with national and international radiological societies from outside Europe and to discuss selected topics of global relevance in radiology at each European Congress of Radiology (ECR). At the ECRs 2013–2016, the relationship between radiology and nuclear medicine, the position of ultrasound in radiology, the relationship of general radiology and subspecialty radiology, and implementation of clinical decision support and imaging referral guidelines in the clinical routine, respectively, were discussed. At ECR 2017, the topic was the position of interventional radiology within radiology.

Representatives of the following radiological societies, usually the president, were invited to this meeting to present the situation in their respective country or region: the American College of Radiology (ACR), Asian Oceanian Society of Radiology (AOSR), Canadian Association of Radiology (CAR), Chinese Society of Radiology (CSR), Colombian Association of Radiology (ACR), Indian Radiological and Imaging Association (IRIA), Inter-American College of Radiology (CIR), International Society of Radiology (ISR), Japan Radiological Society (JRS), Korean Society of Radiology (KSR), Mexican Federation of Radiology and Imaging (FMRI), Radiological Society of North America (RSNA), Radiology Society of United Arab Emirates (RSE), Royal Australian and New Zealand College of Radiologists (RANZCR), Paulista Society of Radiology and Diagnostic Imaging (SPR), Cardiovascular and Interventional Radiology Society of Europe (CIRSE) and European Society of Radiology (ESR). Representatives of several past “ESR meets” countries/societies were also invited to attend the meeting.

The use of high-tech diagnostic imaging procedures has seen double-digit increases annually in the last decade. Likewise, IR has expanded considerably due to the high patient demand for minimally invasive therapies. Nowadays, minimally invasive therapy under imaging guidance is performed in many organs and organ systems, leading to improved patient outcomes, lower morbidity and mortality and is often replacing surgery. It is cost-effective and has proved successful; IR is an integral part of vascular medicine, oncology and many other areas of modern healthcare. It requires specific training, which differs worldwide. IR is also one of the areas of radiology that is most exposed to turf battles. Relations between diagnostic and interventional radiologists are sometimes not harmonic. The ESR believes that the topic of the position of IR within radiology is important and deserved discussion at the International Forum. The representatives of partner societies were asked to present the organisation of IR in their countries/areas, education and training in IR, research in IR, professional status of IR, and relations between IR and general radiology in clinical practice, turf battles, and the percentage of work-time of radiologists spent in IR.

## The situation in North America

*R.L. Ehman* presented for the Radiological Society of North America (RSNA). IR in the USA has become very popular in recent years and is seen as a distinct clinical specialty. The American Board of Medical Specialties approved IR as a primary specialty in 2012. The IR/Diagnostic Radiology (DR) Dual Certificate is one of four primary certificates offered by the American Board of Radiology. The training structure in the USA has moved away from a fellowship model and towards a specialised integrated IR/DR residency. The first 3 years of training are the same as traditional DR residency. Currently 62 programmes exist in the USA, with more expected to be added in the next 5 years. The clinical practice of IR is now very varied in the USA, but there is a trend towards increasing longitudinal patient care. Interventional oncology (IO) has become a major growth area. It started with local palliative ablative techniques for liver malignancies but rapidly expanded to application in other organs. These techniques are now widely available in most hospitals in the USA.

*J.A. Brink*, representing the American College of Radiology (ACR), gave a report on the USA’s perspective on IR residency and on developing a new IR training programme that incorporates the necessary clinical training by increasing the length of IR-specific training without extending the overall length of training or disenfranchising and interfering with DR training, but giving more flexibility to the programmes and the trainees, who can enter from medical school, during DR or after DR, which is a complex but flexible formula. The goals are to allow trainees to be certified in both diagnostic and IR, to bring individuals directly into training from medical school and to continue to bring individuals in from diagnostic radiology. The formula minimises extra training time by utilising change in the diagnostic radiology examination paradigm, and provides options and flexibility. There are two pathways: Integrated Residency (clinical year internship plus five residency years) or Independent Residency (completed internship and diagnostic radiology residency with 2 years of IR residency). All training will be transitioned to this new system and the 1 year IR fellowships will eventually be phased out. In the Integrated Residency programme, the first 3 years are essentially diagnostic radiology residency, with an American Board of Radiology core exam taken after 36 months. The two final years are spent in IR, which qualify for the American Board of Radiology IR/DR certificate. IR Independent Residency requires completion of DR residency. The IR residency programme may be reduced to 1 year for those Diagnostic Radiology residents who complete ESIR (Early Specialisation in Interventional Radiology). J.A. Brink gave a detailed overview of the ESIR structure that requires 12 IR or IR-related rotations and 500 procedures. This means that the DR programmes must have adequate IR facilities and personnel to provide IR training and it allows DR programmes to have an “approved” IR curriculum that would allow DR graduates who complete the ESIR training and satisfy the IR procedural requirements to be eligible to enter directly into the 2nd year of the Independent IR Residency programme. It is expected that in 2020–21 the first Interventional Radiology Independent residents will graduate, while in 2022–23 the first residents that matched to Interventional Radiology Integrated Residency at end of 2017 will finish training.

*W.D. Miller*, representative of the Canadian Association of Radiologists (CAR) presented the situation in Canada, where IR was recognised as a distinct subspecialty of Medical Imaging in 2013. Previously, only neuroradiology and paediatric radiology were recognised. Currently, a new separate exam and a new accreditation programme are under development with the aim to have the first established, accredited programme in 2018. This took years of efforts to achieve. Interventional neuroradiology (INR) exists within neuroradiology, which is also recognised as a distinct subspecialty of radiology. INR is likely to be recognised by a special diploma. INR falls within neuroradiology, but INR training is not funded provincially and INR is not recognised as a separate subspecialty. IR training programmes should become more consistent in quality with the aim to train more clinical skills. In the IR/DR pathway, it is important that interventional radiologists become DRs first. Accredited programmes will be eligible for provincial residency training funding. In Canada, most interventional radiologists work both in interventional and diagnostic radiology, and very few do only IR. IR will be more clinical and interventional radiologists will probably earn less money in the Canadian healthcare system, which is considerably different compared to the USA and many other countries. This may create financial stress in groups and may serve as a disincentive to hire and promote IR, but they will be a visible “value added” to patients and other clinicians. In Canada, IR is essentially all hospital based, in both academic and non-academic centres. Non-vascular work may be performed in few clinics, and the number of spine interventions in clinics is growing. Spine interventions are performed by interventional radiologists, interventional neuroradiologists and other radiologists, but also by orthopaedic surgeons. Although most are hospital-based, the numbers performed in clinical setting are increasing. They can be profitable and there is a huge demand for pain management. Interventional neuroradiology training is completely separate from IR. Sixty percent of INR cases are performed by radiologists and 40% by neurosurgeons. In the majority of large centres, there is a slight INR/IR overlap, while in few smaller centres interventional radiologists do INR work. This is a rather controversial issue and the training is uncertain. There are 18 INR centres in Canada, mostly in large cities, with approximately 50 interventional neuroradiologists. Regarding the public versus private ratio of IR and INR, essentially all centres and procedures are publicly funded and fees for IR and INR in Canada are low compared to imaging. IR and INR are money losers for radiology groups and for medical imaging budgets, but they save money for intensive care units, neurosurgery, surgery, etc.

## The situation in Latin America

The representative of the Inter-American College of Radiology (CIR), *M.Á. Pinochet Tejos*, spoke about the organisation of IR in Latin America, about education and training, research, about professional status and the relationship between interventional and general radiology in clinical practice, about turf battles and the percentage of work time spent in IR. The data presented are based on surveys conducted by CIR in almost all Latin American member countries. IR procedures performed in CIR country members are: image-guided biopsies, visceral interventions (drainage of collections, biliary interventions, nephrostomies, etc.), vascular interventions, neuro-interventions and cardiac interventions. These interventional procedures are performed by radiologists as follows: image-guided biopsies, 92%; visceral interventions, 86%; vascular interventions, 69%; neuro-interventions, 52%; cardiac interventions, 2%. Only 50% of IR procedures are available on a 24 h per day and 7 days per week (24/7) basis. Regarding the inclusion of IR in the syllabus of radiology residency, the procedures in which the residents are trained are: biopsies, fluid collections drainages, and to a very small proportion biliary drainages, nephrostomies and angiographies. Seventy-eight percent of radiology residency programmes include IR, while 22% do not include IR at all. Only 10% of radiology residents choose IR as a main subspecialty for their future professional practice. Seventy-five percent of those surveyed considered residency training in IR and only 25% considered it adequate. Changes proposed are fellowship training, specialisation and more dedication. There is no regulated research done in IR in Latin America. There are six interventional radiologists per 100 diagnostic radiologists in Latin America with an average of 29 interventional radiologists per country (in a range of 1–120). Regarding turf battles, in Latin America there is a constant loss of a space for IR in all areas, mainly due to the lack of training and interest of radiologists themselves. The total duration of the radiology residency programme is 36–48 months, of which 1–4 months are dedicated to training in IR. In conclusion, the main problems from the CIR’s point of view are the small number of interventional radiologists, insufficient dedication, radiation risks, higher working stress, limited training, lack of interest among young radiologists and lack of materials. CIR considers that there is a need to promote training in IR in Latin America, using the system of scholarships for radiologists interested in IR in order to facilitate the access to training centres, and to overcome the legal difficulties for the training.

*B.E. González Ulloa* spoke on behalf of the Mexican Federation of Radiology and Imaging (FMRI). She gave a short overview of FMRI, which was founded in 1974 and currently counts 29 member societies. She reported on the survey carried out in Mexico already mentioned by M.Á. Pinochet Tejos, and mentioned that the FMRI used the same approach as the Inter-American College of Radiology to present comparable data. In Mexico, all IR procedures are available on a 24/7 basis. Regarding the inclusion of IR in the curriculum of radiology fellowship, the procedures in which the fellows are trained are: biopsies (100%), drainage of fluid collections (100%), biliary drainage (50%), nephrostomies (65%), angiography (80%), embolisations (50%), angioplasties/stentings (50%). All radiology fellowship programmes include IR. However, only 7% of radiology fellows choose IR as a main subspecialty for their future professional practice. Research is not always regulated. Only 140 interventional radiologists work in Mexico compared to 4,100 diagnostic radiologists. The percentages of IR procedures that are performed by radiologists in Mexico are as follows: image-guided biopsies, 98%; visceral-interventions, 90%; vascular interventions, 80%; neuro-interventions, 30%; cardiac interventions, none. As in other Latin American countries, Mexicans think that IR training needs to be enhanced by promoting scholarships for radiologists interested in IR and facilitating the access to training centres.

*J.M. Lozano Barriga* presented the situation in Colombia, representing the Colombian Association of Radiology (ACR). Out of 1,300 Colombian radiologists, 1,050 are members of the ACR. The audience was informed about educational centres and medical school, the work time spent in IR, as well as the good cooperation between interventional and general radiology in Colombia. Seventeen postgraduate programmes in Radiology and Diagnostic Imaging exist in Colombia and one training programme in IR (two more are waiting for approval). Residents spend 3–4 months in IR during their residency in radiology. The relationship between IR and DR is good, IR is part of the radiology departments in Colombian hospitals, and general radiologists perform basic interventional procedures, like biopsies, drainages, etc. The majority of interventional radiologists are members of the ACR. Only 75 radiologists in Colombia (6% of ACR members) are full-time interventional radiologists, and approximately 310 (25%) of Colombian radiologists are engaged part-time in interventions. Statistics were shown regarding turf battles in Colombia, and the paper “Who should be doing endovascular surgery?” by E.B. Dietrich was mentioned in this regard, in which each specialty has arguments for its participation in IR procedures. Regarding the peripheral arterial interventions in Colombia, 50% are performed by interventional radiologists, 30% by vascular surgeons and 20% by interventional cardiologists. Seventy percent of neuro-interventions are performed by interventional neuroradiologists, 20% by neurosurgeons and 10% by neurologists. Seventy percent of vascular access procedures are performed by radiologists, and 90% of biopsies and drainages, as well as 90% of haemodialysis fistula interventions. IR can be enhanced with greater dedication to the profession, performing patient care in the office, following standards and protocols, promoting interventional groups in clinics and hospitals, participating in clinical decision meetings at the hospital and working in interdisciplinary groups, and by focusing on residents and training.

*R.A. Mendonça* representing the Paulista Society of Radiology and Diagnostic Imaging (SPR) gave a brief introduction on the radiology societies in Brazil. The Brazilian College of Radiology (CBR) has 13,000 members and 27 affiliated State Societies, among them the SPR. The SOBRICE (Brazilian Society of Interventional Radiology and Endovascular Surgery) and the SBNRDT (Brazilian Society of Diagnostic and Interventional Radiology) are now under the umbrella of CBR. R.A. Mendonça pictured the situation in Brazil with regard to education and training, research, time spent in IR and briefly reported on the differences between interventional and general radiology in Brazil. Radiology itself is recognised as a specialty by the Brazilian Medical Association and one can register on the Regional and/or Federal Medical Council as an interventional radiologist or as a neuroradiologist. According to the law, in principle every Brazilian physician is allowed to act as an interventionalist. There are 16 recognised IR training centres in Brazil, which are certified by SOBRICE and the SBNRDT. The period of training is 2 years full time. The prerequisites are to be accredited in one of the following: radiology, vascular surgery, neurology or neurosurgery. IR is not part of the medical school curriculum. There is, however, some exposure of the topic to the students in very few schools in large centres, with a few classes or optional courses. IR research is relatively new in Brazil, but there is a steady increase in the number of publications in the last years and the description of a pioneer technique of prostate embolisation for benign hyperplasia was made by the University of São Paulo group. In Brazil, most IR services are part of the radiology departments. Increasing numbers of radiologists are looking for IR training due to the growing number of US and CT interventional procedures. Several “not yet officially” recognised training programmes exist for the “non-vascular and non-neuro procedures” with a year-long duration. Simpler IR procedures are still—and should still be—performed by general radiologist, and are therefore directly regulated by the CBR. Regarding turf battles in Brazil, like elsewhere, they exist primarily with vascular surgery and interventional cardiology. While there is some overlap, the range of procedures that IR covers is significantly larger. Some neurosurgery groups are also beginning to train their members to perform IR procedures without involving radiologists. IR practitioners usually dedicate themselves to IR exclusively, with the exception of minor procedures performed by general radiologists. To sum up, IR is a relatively new area in Brazil; there is a continuous effort to keep IR within radiology, but the risk exists that IR will be absorbed by other specialties.

## The situation in the Arab countries

*A. Alremaithi* spoke on behalf of the Radiological Society of Emirates (RSE), and gave some background information on the United Arab Emirates (UAE) and the RSE, which was established in 2008 and acts under the umbrella of the Emirates Medical Association (EMA). IR is recognised as a medical subspecialty by the UAE health authorities and most interventional radiologists do both DR and IR. There are currently no dedicated training programmes in IR in the UAE; radiologists go abroad for training, and there is a lack of human resources in the country. Rotation in IR during the residency lasts 4 months. In medical school, students spend 2 months in the radiology department, but get little or no exposure to IR. There is a good connection between interventional and general radiology in clinical practice. UAE radiologists participate in research. In terms of time spent in IR, in government hospitals it is 70–100%, and in private service 30–50%.

*T. El-Diasty*, president of the Egyptian Society of Radiology and Nuclear Medicine (ESRNM), and representative of the past ‘ESR Meets’ country Egypt, briefly reported on the situation in Egypt during the discussion following A. Alremaithi’s presentation. Egypt counts 800 general interventional radiologists and 200 subspecialised interventional radiologists. A fellowship programme on IR has already been implemented. The main threat for IR is that many non-radiologist medical doctors are performing it.

## The situation in India

*B. Ahuja* spoke on behalf of the Indian Radiological and Imaging Association (IRIA). While therapeutic interventions in India are provided in dedicated centres, interventions under ultrasound (US) guidance are performed directly by radiologists in many hospitals. The number of institutions performing IR procedures has grown and today more than 70,000 procedures are performed in 65 institutions, mostly in larger cities. Dedicated vascular interventions are performed only in a handful of radiology departments across the country. Education programmes in India were presented, including the Annual Congress of IRIA with dedicated sessions on IR, the Annual Conference of ISIVR (Indian Society of Vascular and Interventional Radiology), and CME events. ISVIR has 500 members, which reflects the number of interventional radiologists in India and the lack of manpower is hindering the growth of IR. Most medical schools do not have the manpower or the infrastructure to train interventional radiologists. IR represents the face of radiology in India because this is the only branch where radiologists have direct contact to the patients, and dedicated beds allotted to IR are available in the hospitals. However, India is still facing many challenges in this sector; in particular, with regard to training, lack of interventional materials and cost issues. India is primarily working on promoting the use of interventional procedures to further institutions and radiologists in private practices, and improving education and training in medical schools and specialised institutes. Significant turf issues exist with other specialties. All coronary-related work is done by cardiologists, who are also gradually doing a lot of carotid, peripheral vascular interventional work. Neurologists and neurosurgeons in some institutions have started doing neuro-interventions and vascular surgeons doing vascular interventions. For IR to grow and remain in the hands of radiologists, a direct relationship of radiologist and patient is a must, and the IR departments have to have their own beds and control over admissions and clinical management of the patients. A few main Indian centres have already started having three to four dedicated beds in wards for IR.

## The situation in Japan and Korea

*H. Honda*, on behalf of Japan Radiological Society (JRS), gave a general report on the organisation of IR in Japan, education, training, research, turf battles and time spent in IR. The JRS has good relationships with the Japanese Society of Interventional Radiology (JSIR), which was first part of JRS and became independent in 2016. JSIR has also started an IR expert nurse training system in 2009. Further, 95% of JSIR members are also JRS member radiologists. Educational guidelines of JRS and JSIR are to be followed and 250 training institutes in Japan met the requirements for certification in IR, meaning that they need to have at least one board certified interventional radiologist and more than 200 cases of IR every year. Obtaining the Board of Interventional Radiology certification requires to be a Board-certified diagnostic radiologist and to be trained for two more years in IR, as well as to perform more than 200 cases per year in the training institute. Education includes passing oral examinations and writing papers. The Board certification of a diagnostic radiologist (JRS) after 5 years of training is required to obtain the Board of Interventional Radiology (JSIR) certification. The JSIR now has 964 Board-certified members, 93% of which are men and 7% women. Moreover, IR research in Japan is very active; two scientific journals are published by the JSIR, namely the *Journal of JSIR* (in Japanese) and *Interventional Radiology* (in English) and many papers are published in the U.S. and European journals (in 2016, 82 papers from Japan were published in *JVIR* and *CVIR*).

The number of pure interventional radiologists in Japan is not so large because radiologists generally must cover both diagnosis and intervention in Japan. However, the amount of interventional engagement for a particular radiologist varies a lot and depends on the person and/or institute. The salary of radiologists in Japan is quite average and does not depend on the type of work performed. As a result, Japanese radiologists cover both diagnostic and IR work without any conflicts.

*S.H. Kim*, representing the Korean Society of Radiology (KSR), gave some background information on the Korean Society of Interventional Radiology (KSIR), previously named Korean Society of Cardiovascular and Interventional Radiology (KSCVIR). With over 270 official members, KSIR promotes many activities in education and scientific research on both domestic and international level. KSIR and KSR also collaborate in the organisation of the IR subspecialty during KSR’s congress, KCR. Regarding education, radiologists are first exposed to IR during their residency rotation. After 4 years of residency, those who want to be trained and Board-certified in IR undergo fellowship training in more than 50 training centres. Among the different educational programmes, the IICIR (International Intensive Course for Interventional Radiology) takes place annually in Seoul. This course is addressed to Korean residents as well as residents from the Asia-Pacific region, and also aims to improve the cooperation with IR societies from this region. KSIR also organises practical workshops and encourages active participation of its members in scientific research by organising annual scientific meetings and awarding the “best scientific presentation”. Korea is not an exception when it comes to turf battles. Since IR is incorporated in the radiology department, IR and general radiology are closely integrated in both academic and administrative aspects.

## The situation in China and the Asia-Oceania region

*Y. Wang* spoke on behalf of K. Xu, President of the Chinese Society of Radiology (CSR), about IR in China. IR is the biggest subgroup within the Chinese Society of Radiology. IR is a new field for radiologists, and interventional radiologists are actively involved in patient consultation, guidance and treatment, beyond imaging. Intervention provides an alternative to drug therapy and surgery with the advantage of being minimally invasive as it is precise, effective and repeatable. She also mentioned the advantages of technologies in this field which enable adequate visibility, precise localisation and increased accuracy, allowing a better targeting of the lesion without radiation exposure of the patient or the staff. In China, not only interventional radiologists but also cardiologists, vascular surgeons, neurosurgeons, oncologists and other physicians are involved in intervention, and the procedure volume grew at faster rates among other doctors and physicians than among radiologists. The future of IR is in further expanding the capabilities of IR practices, heading towards subspecialisation and developing skills-based medical training.

*Y.-H. Chou* spoke on behalf of the Asian Oceanian Society of Radiology (AOSR). He presented for the 26 countries (including Hong Kong) which are part of the Asian-Oceanian region and which are grouped in four main clusters—namely, North East Asia, South Asia, Japan and South Korea, Pacific and ASEAN. Among the 24 member societies of AOSR, the radiological societies of China, Taiwan, Hong Kong, India, Korea, Singapore, Australia and New Zealand, and in particular Japan, are very advanced in the field of IR with regard to organisation, education, research, professions and work time. However, the shortage of radiologists and, consequently, increasing workload are noted in almost all countries; the lack of new technologies and of knowledge and quality standards in general, as well as the presence of image-guided interventions, training and imaging services are still some of the major issues in about half of the countries. Turf battles exist, like elsewhere.

## The situation in Australia and New Zealand

*G. Slater* represented the Royal Australian and New Zealand College of Radiologists (RANZCR) that has about 4,000 members, including students, trainees and radiation oncologists. All radiologists are trained to perform simple IR procedures, like biopsies, drainages and nephrostomies, while complex interventions are restricted to members with subspecialty training. RANZCR provides support to two affiliated subspecialist groups, IRSA (Interventional Radiology Society of Australia) with 250 members and ANZSNR (Australian and New Zealand Society of Interventional Neuroradiology) with 60 members, mostly radiologists, but also a few interventional neurosurgeons and neurologists. A major advance has been the development of an IR Committee, which was established by the RANZCR board in 2016, focusing on the training, certification, subspecialty recognition and practice of IR. After certifying with RANZCR one needs an additional 2 years of training for IR and 3 years for interventional neuroradiology. Regarding IR in medical schools, some universities have a curriculum in clinical radiology and IR may be included, but there is no formal national programme. Australian interventional radiologists are included in several research trials, especially in areas of stroke management and vertebroplasty. Turf battles with vascular surgeons, cardiologists and other specialities are present as elsewhere. Very few Australian and New Zealand radiologists are full-time interventional radiologists or interventional neuroradiologists.

## Position of the International Society of Radiology (ISR)

The International Society of Radiology’s (ISR) perspective was provided by *R. García-Mónaco*, who announced that the main goal of the ISR is to facilitate the global endeavours of ISR’s member organisations in order to improve patient care and population health through medical imaging. Most of the ISR member countries have already presented their perspective during this forum, showing similarities and some differences between them. The situation in African countries is probably not very different with regard to the aspects presented in this forum. The question is whether IR should be performed within or outside radiology, and the major problem is the heterogeneity among the different countries. ISR has not defined a position so far on the matter, the subject has not been addressed by the society and no actions or questions were asked by its members or by the World Health Organisation. The ISR is mainly devoted to help or act in emerging areas of the world, and not to overlap with national or continental societies’ endeavours. As a global facilitator, the ISR could assist if required by members. His personal opinion is that IR should be regarded as a true subspecialty of radiology with specific training, full time activity and commitment to patient care. However, in countries where IR is not well developed, minor procedures could be technically well performed by part time radiologists.

## The situation in Europe

*E. Brountzos* on behalf of the Cardiovascular and Interventional Radiological Society of Europe (CIRSE) presented CIRSE’s position. He emphasised that IR is a successful subspecialty of radiology, due to the high patient demand for minimally invasive therapies, lower morbidity and mortality of IR procedures, cost-effectiveness and proven success. The current challenges for IR are competition from other specialties, lack of clinical control and of robust subspecialty training, as well as the lack of a strong undergraduate education and of public awareness. He suggested that the only way to ensure a future for IR is to continuously evaluate current structures, issues and challenges, not to lose what has been achieved. IR recognition as subspecialty in radiology is important, dedicated IR training pathways are mandatory, clinical responsibility (longitudinal patient care) both in an inpatient and outpatient set-up is crucial, as well as recruitment of young doctors to IR and increasing public awareness. A survey on clinical practice in IR among CIRSE members has shown that in Europe turf battles and lack of clinical practice in IR represent a major threat in most countries. In only 27% of the departments that participated in the survey, inpatient beds exist that are dedicated to IR. The large majority of these beds are located in surgical or medical wards and less than 25% in the dedicated IR hospital wards. E. Brountzos also reported that CIRSE and ESR have been cooperating very closely with regard to IR in the basic levels of the European Training Curriculum for Radiology (Level I and II), the European Curriculum and Syllabus for Interventional Radiology [its revised second edition 2017 provides indications to do the European Diploma in Radiology (EDiR) examination, and specifications for the EBIR], and the certification in IR through the European Board of Interventional Radiology (EBIR). EBIR was established in 2010, endorsed by ESR and UEMS/IR Division. Sixteen EBIR examinations were held to date, with three additional planned in 2017. One EBIR exam per year is held in Australia/New Zealand in cooperation with IR and the radiology societies IRSA (Interventional Radiology Society of Australasia) and RANZCR (Royal Australian and New Zealand College of Radiologists). Currently there are over 500 EBIR holders. A solid pathway for young interventional radiologists has also been developed by CIRSE. This includes the IR Curriculum for medical students and the European Student Programme for medical students, the European Trainee Forum and the European Curriculum and Syllabus for IR dedicated to IR trainees. Changes and improvements were made of the revised second edition with the increased focus on clinical practice and safety. Also, a separate section on Interventional Oncology has been added. Obsolete therapies (e.g. fallopian tube recanalisation) have been excluded and new therapies (e.g. prostate artery embolisation for benign prostate hyperplasia) have been added.

*K. Riklund* on behalf of the European Society of Radiology (ESR) presented an overview of the ESR activities with respect to IR. About 17% of all ESR members in 2016 indicated an interest in IR. In the ESR Statutes, radiology is described as ‘diagnostic and interventional radiology, biomedical, molecular and functional imaging’. K. Riklund reported on the ESR’s endorsement of subspecialty diploma, and that IR is included in the ESR European Training Curricula [U-Level, Level I-II, Level III – Subspecialisation (*see* https://www.myesr.org/education/training-curricula)]. A new revised version of the ESR Curriculum for Undergraduate Radiological Education (U-Level) has been online since March 2017, and the Level III is being finalised with the Subspecialties and Allied Sciences Societies and will be published online in autumn. She clearly pointed out that also in the ESR’s point of view, IR should remain a subspecialty of radiology.

## Discussion

IR has established itself globally as an important subspecialty of radiology. It is expanding because of the high patient demand for minimally invasive therapies performed under imaging guidance. IR has proven success in improving patient outcomes, reducing in-hospital stay, reducing morbidity and mortality of treatment and replacing surgery in many organs and organ-systems and in many areas of modern healthcare. It can be assumed that the current interventional procedures and future developments in image-guided interventions will dominate treatment in medicine in the future. However, despite the many successes of IR over last years and decades, public awareness about the importance of IR is still quite low and should be increased.

IR requires specific training, which differs considerably worldwide. The training programmes need to include all modern IR procedures, which are changing and expanding rapidly. It needs to incorporate the necessary clinical training, and to increase the length of IR-specific training without extending overall length of training or interfering with diagnostic radiology (DR) but giving more flexibility to the programmes and the trainees. Training curricula of ESR and CIRSE are very important in this respect [1, 2].

All participants in the Forum agreed that the future of IR, as a well-defined and important subspecialty, is within radiology. So the goals of education are to allow trainees to be certified in both diagnostic and IR. It was shown in the Forum that in the majority of countries IR procedures are still performed part-time, combined with DR, and only a minority of radiologists dedicate themselves exclusively to IR. This varies greatly depending on the region and number of practicing interventional radiologists and diagnostic radiologists. As pointed out by the Latin American representatives, it seems prudent that general radiologists perform only minor IR procedures, like biopsies and drainages, and thereby cover the demand for treatments in hospitals as far as possible, and that subspecialisation in IR should be dedicated to more difficult procedures. This way, radiologists would protect themselves from other subspecialties performing minor procedures.

The major issue emerging from the presentations is a low level of interest in IR in many areas of the world, which could primarily be related to the problem of turf battles. But there is also a threat preventing medical students from becoming diagnostic radiologists, namely the fear to lose their jobs through new technologies, such as teleradiology or other computer-based systems. This may encourage medical students to enter IR in lieu of diagnostic radiology. The way to attract those who are interested in becoming interventional radiologists is by improving the educational programmes and creating new posts in hospitals for IR and investing into IR divisions within radiology departments. Also, more attractive salaries for interventional radiologists would improve the chances of bringing students closer to IR. The issue of salaries varies considerably between different countries.

Turf battles in IR are common in many countries. They are especially prominent in the area of endovascular procedures and treatment of peripheral vascular disease; boundaries are being crossed, and conflict and competition have become inevitable. Vascular surgeons consider peripheral vascular disease as their traditional domain, because they are the only ones capable of total care, from diagnosis through procedure and follow-up. Radiologists consider that endovascular procedures are historically IR territory and that surgeons are ill-trained for percutaneous approaches, while cardiologists have no experience in the treatment of peripheral vascular disease. Cardiologists think that an artery is an artery, a balloon is a balloon, and it does not matter in which artery you are placing the stent; they have patients, the laboratories and the catheter skills [3, 4]. To preserve and keep IR within radiology, it is necessary to focus more on direct and longitudinal patient care. Having beds dedicated to IR within radiology department is very important to increase clinical involvement of interventional radiologists. Although beds dedicated for IR within radiology departments are still very uncommon in all countries and continents, we should support such a system whenever possible, because it is a very important strategic point for the future development of IR.

Another important issue is the relationship between INR and IR with regard to stroke treatments; concretely, whether stroke treatment should be also performed by interventional radiologists, given the limited number of interventional neuroradiologists who should perform such treatments in the first instance. There was general agreement that interventional radiologists should be trained in order to be able to perform this treatment, and the CIRSE has already established European School of Interventional Radiology courses across Europe with this scope.

The concept of “value-based healthcare” has emerged as a framework for achieving better results, considering factors that matter to patients, while optimising the cost of care delivery within the health system, and the ESR is involved in evaluating the position of radiology in this concept [5]. Value is defined as health outcomes achieved for patients relative to the costs. Value depends on the results of care and is measured by reference to the results obtained, and not on the volume of services delivered [6]. Within this concept, payments are assigned according to the outcomes of a given episode of care, and good outcomes have to be obtained in the most efficient way to achieve a reduction in their costs. Radiologists play a fundamental role in the diagnostic process of modern healthcare delivery, but are often considered as factories producing imaging examinations, with attention focused only on the number of procedures performed. Diagnostic radiology work is considered as a chain of processes and their results, the diagnoses, are not regarded as an outcome. In the clinical projects implementing value-driven programs that have been developed, diagnostic radiology has been simply considered as a cost and measured as such. The diagnosis, and how it has been possible to reach it, has not been regarded as the first important result of the entire episode of care [7]. IR is different, since the results of interventional procedures can be considered directly as outcomes under existing models. They fit perfectly within the value-based healthcare framework: patients’ preferences can be assessed, costs can be measured and “value” calculated and compared with that of other therapeutic procedures. However, IR procedures and their outcome are linked to the work of the other doctors who precede and follow the intervention and are thus subject to quality of both the referrals and follow-up. Furthermore, no “value” can be calculated regarding the correct choice and quality of the diagnostic examinations performed to decide on the feasibility of the procedure and to guide it, and these are often performed immediately before the intervention, in the same session, and often by the same radiologist. Thus, metrics about these must also be developed.

## Conclusions

Recognition of IR as a subspecialty in radiology is important. Dedicated IR training pathways are mandatory, as well as increasing clinical responsibility (longitudinal patient care) that are crucial to preserve imaging-guided minimally invasive treatment procedures within the umbrella of radiology and position IR in the turf battles with other specialties. Having beds dedicated to IR within the radiology department is very important to increase clinical involvement of interventional radiologists. Measures to increase the recruitment of young doctors to IR are needed and increasing public awareness should be achieved. IR should stay within radiology and specificities of IR have to be observed.
